# 13-Year-Old with Cryptic Abdominal Pain

**DOI:** 10.5811/westjem.2014.11.24429

**Published:** 2014-12-05

**Authors:** Stephanie Spring, Richard Anderson, Jestin N. Carlson

**Affiliations:** * Saint Vincent Health System, Department of Emergency Medicine, Erie, Pennsylvania; † Unviersity of Pittsburgh School of Medicine, Department of Emergency Medicine, Pittsburgh, Pennsylvania

A 13-year old female patient presented to the emergency department (ED) with four days of intermittent non-radiating, left upper quadrant pain, associated with non-bloody, non-bilious emesis and decreased appetite. The patient had been evaluated by a gastroenterologist three months prior for abdominal pain. At that time an esophagogastroduodenoscopy revealed a trichobezoar in the stomach too large for endoscopic removal. Elective surgical removal had been offered to the patient, although surgery had not yet been scheduled.

Vital signs were within reference limits for the patient’s age. The patient had tenderness throughout the left upper quadrant, however there was no guarding or rebound tenderness. The patient underwent computed tomography (CT) of the abdomen and pelvis, which confirmed the thichobezoar in the stomach and intussusception of the bowel (Figures 1 and 2) consistent with Rapunzel Syndrome.

## DISCUSSION

**Figure 1:**
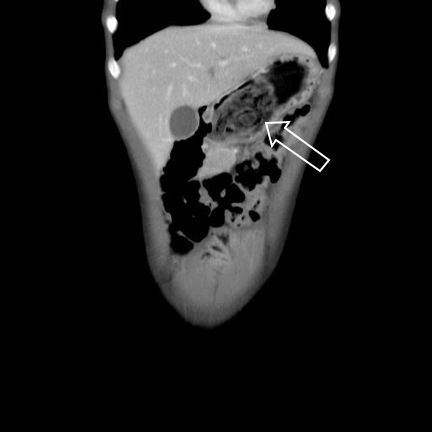
Bezoar in the stomach.

**Figure 2:**
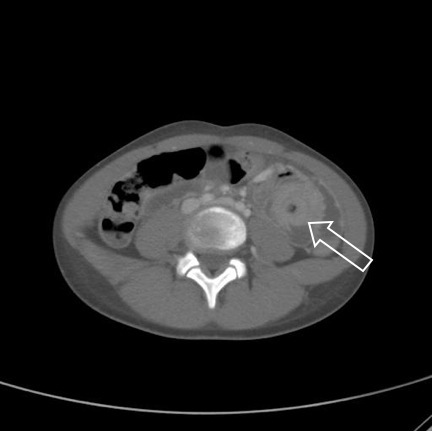
Target sign signifying intussusception. The arrow points to the tail of the bezoar acting as a lead point for the intussusception.

Rapunzel Syndrome, named after the Brothers Grimm’s fairy tale princess with long hair by the same name, is a rare disorder resulting from trichobezoar formation and subsequent extension of the tail of the bezoar into small bowel associated with trichophagia (Figure 1).^1^ The tail of the hair acts as a lead point, causing intussusception of the bowel (Figure 2). Bezoars are rare, occurring in less than 1% of patients undergoing upper gastrointestinal endoscopy and only a small number of patients have been reported with Rapunzel Syndrome.^1^–^3^ While many patients may undergo CT scans, ultrasound and plain radiographs have also been used to establish the diagnosis.^4^,^5^ Management includes removal via endoscopy or elective surgery although complications including gastric perforation has been reported.^6^,^7^ The patient was transferred to a pediatric hospital where she underwent an exploratory laparotomy including bezoar removal from both the stomach and jejunum. Over the next 48 hours the patient’s diet was advanced and was discharged home on post-operative day five.
